# Vapor-Phase Polymerization of Polypyrrole on Carbon Cloth: Simultaneous Tuning of Surface Resistance and Dielectric Permittivity for High-Performance Flexible RF Antenna Electrodes

**DOI:** 10.3390/polym18172124

**Published:** 2026-08-31

**Authors:** Seung Ji Kim, Se Eun Lee, Kyein Kim, Keun-Young Shin

**Affiliations:** 1 Department of Materials Science and Engineering, Soongsil University, 369 Sangdo-ro, Dongjak-gu, Seoul 06978, Republic of Korea; tmdwl0130@soongsil.ac.kr (S.J.K.); dltpdms8197@soongsil.ac.kr (S.E.L.); kyeinee@soongsil.ac.kr (K.K.); 2 Department of Convergence of Energy Policy and Technology, Soongsil University, 369 Sangdo-ro, Dongjak-gu, Seoul 06978, Republic of Korea

**Keywords:** carbon cloth, polypyrrole, vapor-phase polymerization, flexible RF antenna, complex permittivity, radiation efficiency

## Abstract

Carbon cloth is a lightweight and mechanically robust fibrous substrate with strong potential for value-added flexible electronic applications. In this study, carbon cloth/polypyrrole (CC/PPy) composites were fabricated via vapor-phase polymerization (VPP) and evaluated as radiating electrodes for flexible monopole patch RF antennas. By varying the polymerization time, the surface resistance and complex permittivity of the CC/PPy composites were systematically tuned, enabling simultaneous optimization of electrical conductivity and dielectric response. Among the prepared samples, the composite polymerized for 20 s exhibited the most balanced properties, with a sheet resistance of 1.86 Ω/sq, a real permittivity (ε′) of 5.78, and an imaginary permittivity (ε″) of 0.23. When applied as the antenna electrode, this material delivered a return loss of −34.81 dB, a radiation efficiency of 84.32%, a peak gain of 3.30 dBi, and a peak directivity of 3.91 dBi at 1.74 GHz. In addition, the antenna maintained stable performance after 5000 bending cycles, demonstrating excellent mechanical durability. These results show that simultaneous control of surface resistance and dielectric properties is critical for high-performance flexible RF electrodes and provide a practical surface-functionalization strategy for upgrading carbon-cloth-based fibrous materials into value-added electronic products.

## 1. Introduction

As wireless communication technologies continue to advance, antennas play a critical role as core components that transmit and receive wireless signals in the form of electromagnetic waves. Accordingly, the demand for flexible and lightweight RF antennas has rapidly increased across a wide range of applications, including wearable electronics, the Internet of Things (IoT), and vehicular communications. In these applications, flexible electrode materials that can operate reliably under device deformation, bending, and changes in the external environment are essential.

Antenna performance is commonly evaluated in terms of resonance frequency, impedance-matching characteristics, and radiation efficiency. In particular, the electrical conductivity of the electrode material strongly affects conductor loss, thereby significantly influencing antenna efficiency and overall performance [1]. In addition, the dielectric properties of the electrode material can affect the resonance condition and input impedance, which in turn governs impedance matching [2]. Conventional RF antenna electrodes have typically employed metal thin films such as aluminum or copper, which offer high electrical conductivity. However, metal electrodes are heavy and mechanically rigid, limiting their applicability to flexible devices. They are also susceptible to corrosion, resulting in limitations in long-term stability [3].

To address these issues, various carbon-based materials have been explored as alternative electrode candidates. Among them, carbon cloth (CC) has attracted attention as a flexible electrode substrate owing to its woven three-dimensional fibrous network, which provides multidirectional current pathways, and its rough surface with a large surface area, which is advantageous for improving interfacial adhesion. CC is also lightweight, flexible, and mechanically durable, enabling structural integrity under repeated deformation, which is beneficial for wearable applications [4]. Nevertheless, CC exhibits lower electrical conductivity (~10^3^–10^4^ S/m) than metals, and additional contact resistance at fiber junctions can further increase conductor loss, limiting the antenna performance of pristine CC electrodes.

To overcome these limitations, a small number of studies have reported the use of CC in combination with other materials as antenna components. For example, epoxy-impregnated CC has been utilized as a conductive element, and CC combined with polydimethylsiloxane (PDMS)–ceramic composites has been employed as a microstrip ground plane [5,6]. CC has also been explored as a supporting substrate for graphene-based patches [7]. However, these approaches generally employ CC as a supporting component rather than the primary radiating electrode, and studies that directly utilize CC as a radiating element remain limited. Moreover, prior work has mainly focused on improving electrical conductivity to reduce conductor loss, while the role of complex permittivity, which governs both resonance conditions and impedance matching through its real (ε′) and imaginary (ε″) components, has received comparatively little attention. Systematic investigations addressing the simultaneous optimization of conductivity and dielectric properties for carbon-based flexible RF antennas therefore remain scarce. Accordingly, a key challenge is to functionalize CC in a way that compensates for its electrical limitations without sacrificing its flexibility and fibrous structural characteristics.

Conducting polymers offer tunable electrical properties depending on their doping state and can be synthesized through relatively low-temperature processes. Among them, polypyrrole (PPy) is a representative conducting polymer with a conjugated backbone that provides high electrical conductivity (up to ~10^3^ S/cm) and improved mechanical compliance compared with metal thin films, owing to its lower susceptibility to cracking [8,9]. However, most PPy-based flexible electrode studies rely on solution polymerization, which often makes it difficult to achieve uniform PPy coating on complex three-dimensional substrates [10]. Moreover, electrical properties can fluctuate due to residues from washing and drying steps, and degradation in long-term stability has also been reported.

To overcome these limitations, vapor-phase polymerization (VPP) has attracted considerable attention as an alternative route for depositing polypyrrole (PPy) on carbon-based flexible substrates. Previous studies have shown that vapor-phase-grown PPy coatings on carbon cloth and carbon fibers can improve electrical properties, mechanical flexibility, and electrochemical performance, leading to their extensive investigation in flexible supercapacitors and related energy-storage devices [11,12,13]. Compared with conventional solution polymerization, vapor-phase deposition enables polymerization directly on the substrate surface and is advantageous for forming PPy coatings on porous fibrous architectures while reducing solvent-related byproducts. In addition, the deposition conditions can be systematically controlled, allowing tunable growth behavior of the PPy layer [14,15].

Despite these advantages, prior studies have mainly focused on electrochemical energy-storage applications. The use of vapor-phase-grown PPy-coated carbon cloth as the primary radiating electrode for flexible RF antennas has been rarely explored. Moreover, previous studies have largely emphasized conductivity enhancement or electrochemical functionality, whereas the simultaneous tuning of surface resistance and complex permittivity, which is critical for impedance matching and radiation efficiency in RF antennas, has received little attention. In this regard, the present study aims to address an application area and materials-design perspective that have not been sufficiently investigated in previous vapor-phase-grown PPy/carbon cloth systems.

In this work, CC/PPy composites were fabricated via a simple VPP process and directly employed as the primary radiating electrode of a flexible monopole patch RF antenna. By varying the polymerization time (10, 20, and 30 s), the surface resistance and complex permittivity of the CC/PPy composites were systematically tuned to identify an optimized condition. The morphology and elemental composition of the composites were characterized using field-emission scanning electron microscopy (FE-SEM) and energy-dispersive X-ray spectroscopy (EDS), while chemical bonding and constituent species were examined by Fourier transform infrared (FT-IR) spectroscopy, Raman spectroscopy, and X-ray photoelectron spectroscopy (XPS). Antenna performance was evaluated in terms of return loss (RL) and radiation efficiency. The results highlight the effectiveness of the proposed strategy, simultaneous optimization of conductivity and dielectric properties, for realizing high-performance, flexible RF antenna electrodes based on CC.

## 2. Experimental Section

### 2.1. Materials

CC was purchased from Wizmac (Daejeon-si, Republic of Korea). Pyrrole (reagent grade, 98%) and ammonium persulfate (APS; ACS reagent, ≥98%) were purchased from Sigma-Aldrich (St. Louis, MO, USA). Deionized (DI) water was used throughout all experiments.

### 2.2. Fabrication of CC/PPy Composites via VPP

CC was cut into 1 cm × 1 cm pieces and plasma-treated at 200 W for 30 s. A 5 wt% APS solution was prepared by dissolving 0.525 g of APS in 10 mL of DI water, and the CC was immersed in the solution for 10 s. After APS treatment, the CC was directly used for VPP without an additional drying step and placed in a glass Petri dish with a diameter of 10 cm. Subsequently, 1 μL of pyrrole monomer was introduced into the dish as the vapor-phase monomer source. A second glass Petri dish with a diameter of 8 cm was inverted and used as a cover, and a constant load of approximately 1 kg was applied to the upper dish to maintain a consistent covered configuration during polymerization. The assembly was then heated in an oven at 110 °C. The polymerization time was varied to 10 s, 20 s, and 30 s, and the resulting samples were denoted as CP1, CP2, and CP3, respectively. After polymerization, the samples were dried at 50 °C for 12 h.

### 2.3. Fabrication of CC/PPy Composite-Based Monopole Patch RF Antenna

A CC/PPy composite (1 cm × 1 cm) was used as the radiating electrode of a monopole patch RF antenna. The antenna was directly connected to a Sub-Miniature Version A (SMA) connector through a feed line without an external matching network, and a rectangular copper plate (≥120 mm × 100 mm) was used as the ground plane to provide a stable and reproducible reference structure for comparing the RF characteristics of pristine CC and the CC/PPy radiating electrodes. The ground-plane dimensions and feed configuration were maintained identically for all samples to ensure consistent measurement conditions during antenna characterization. To investigate the effect of polymerization time on the electrical properties of the electrode, the PPy polymerization time was systematically varied to 10 s (CP1), 20 s (CP2), and 30 s (CP3), and the surface resistance of the pristine CC and the resulting CC/PPy composites was measured and compared.

### 2.4. Characterization

The surface morphology was examined by FE-SEM (Carl Zeiss GEMINISEM 300, Jena, Germany). The molecular structure of the composites was characterized by FT-IR (Bruker VERTEX70, Ettlingen, Germany) and Raman spectroscopy (Renishaw Raman microscope, Wotton-under-Edge, UK). Elemental composition and spatial distribution were analyzed by EDS and elemental mapping using an EDS detector (Bruker Nano GmbH, Berlin, Germany) attached to the FE-SEM. The chemical composition and bonding states were investigated by XPS (PHI 5000 VersaProbe, Ulvac-PHI, Inc., Chigasaki, Japan) with monochromated Al Kα radiation. The surface resistance was measured using a four-point probe system (Loresta-GX MCP-T700, Mitsubishi, Tokyo, Japan). The RL (S11) was evaluated using a Keysight E8362B PNA network analyzer (Keysight, Santa Rosa, CA, USA) over the frequency range of 1–3 GHz, with impedance values normalized to 50 Ω and plotted on a Smith chart. The radiation pattern and efficiency were measured in an anechoic chamber at the Electromagnetic Wave Technology Institute of Korea using a vector signal generator (E4438C, Agilent Technologies, Santa Clara, CA, USA), a base station simulator (E5515C, Agilent Technologies), and a WiBro/WiMAX communication tester (CMW270, Rohde & Schwarz, Munich, Germany). Dielectric properties were characterized using an LCR meter (Keysight E4980AL). Mechanical bending tests were performed using a motorized force test stand (Imada MH2-500N, IMADA Co., Ltd., Toyohashi, Japan).

## 3. Results and Discussion

**Figure 1:**
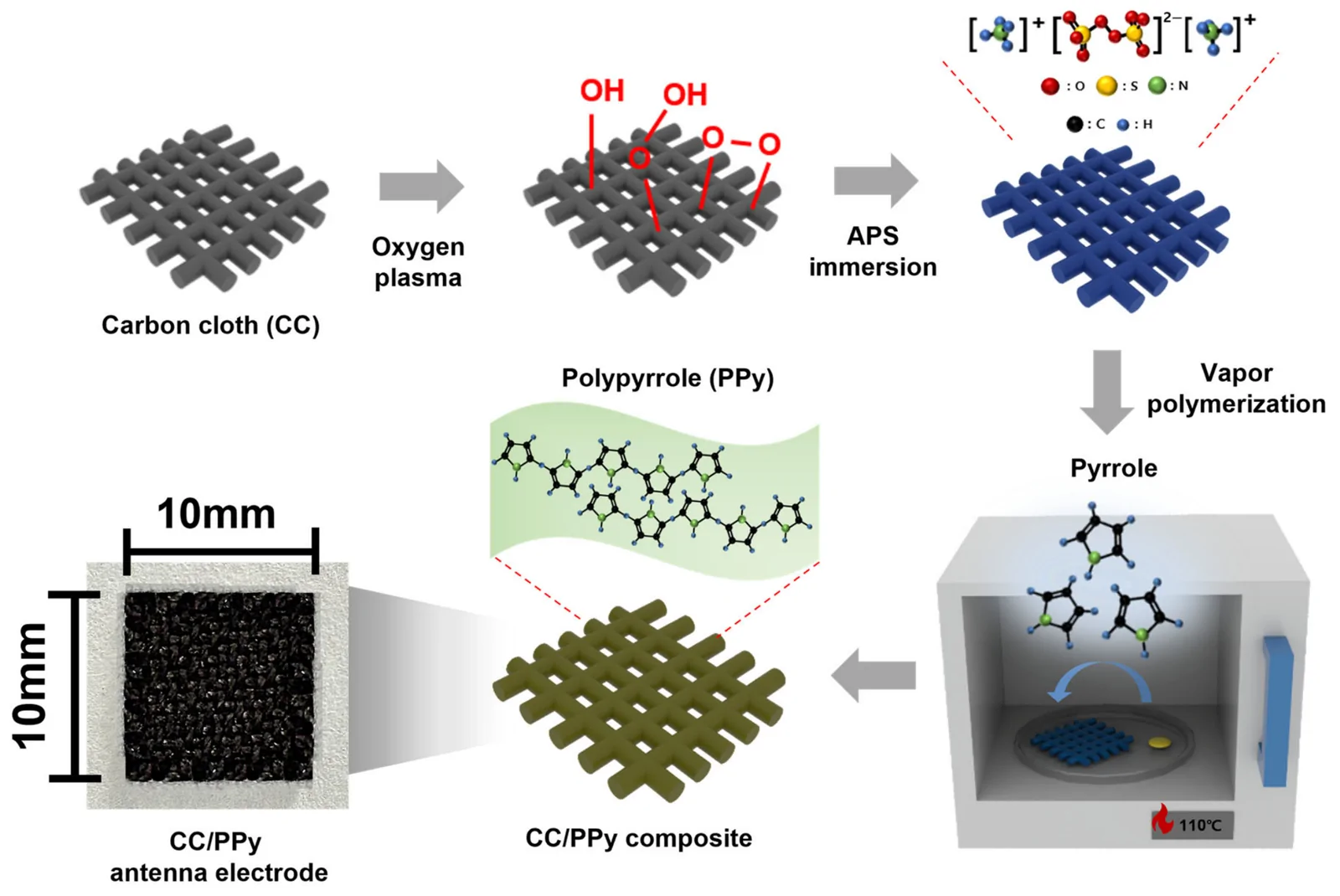
Schematic illustration of the fabrication process for a CC/PPy antenna electrode. CC was first plasma-treated and then immersed in an APS solution to incorporate the initiator. Pyrrole monomer was subsequently vapor-phase polymerized at 110 °C to form the CC/PPy composites (10 mm × 10 mm).

Figure 1 schematically illustrates the fabrication procedure of the CC/PPy composite-based antenna electrode via plasma surface treatment, APS immersion, and VPP. First, the intrinsically hydrophobic CC was subjected to plasma treatment to increase surface energy and wettability, rendering the surface hydrophilic and introducing oxygen-containing functional groups that enhance surface reactivity. This promotes adsorption of the APS initiator, thereby facilitating polymerization initiation [16]. Next, the plasma-treated CC was immersed in an APS solution to ensure sufficient infiltration of APS not only onto the outer surface but also into the inter-fiber voids and the interior of fiber bundles. The improved hydrophilicity enhances solution penetration, resulting in a more homogeneous initiator distribution throughout the woven structure and thereby promoting uniform PPy formation. In this study, the APS-immersed CC was directly subjected to VPP without a separate drying step. This simplified process was adopted to reduce the possibility of local oxidant redistribution or crystallization during drying within the porous fibrous structure of CC. During VPP, heating at 110 °C allows pyrrole vapor to diffuse into the APS-treated CC structure and initiate oxidative polymerization, while also facilitating rapid removal of residual solvent at the early stage of the process. In addition, APS immersion time, pyrrole dose, and APS concentration were preliminarily examined (Figure S1), and the final conditions were selected to achieve low surface resistance while reducing APS consumption and minimizing the possible influence of residual oxidant species.

The resulting PPy grows along the fiber surface, forming a conductive thin film. Notably, PPy formation is not confined to the outer surface; polymerization can propagate into the fiber bundles and the fabric network along APS-infiltrated regions, enabling conformal formation throughout the 3D architecture [17]. The resulting CC/PPy composite was employed as the antenna electrode, and the electrode size was fixed at 10 mm × 10 mm, identical to that of pristine CC.

**Figure 2:**
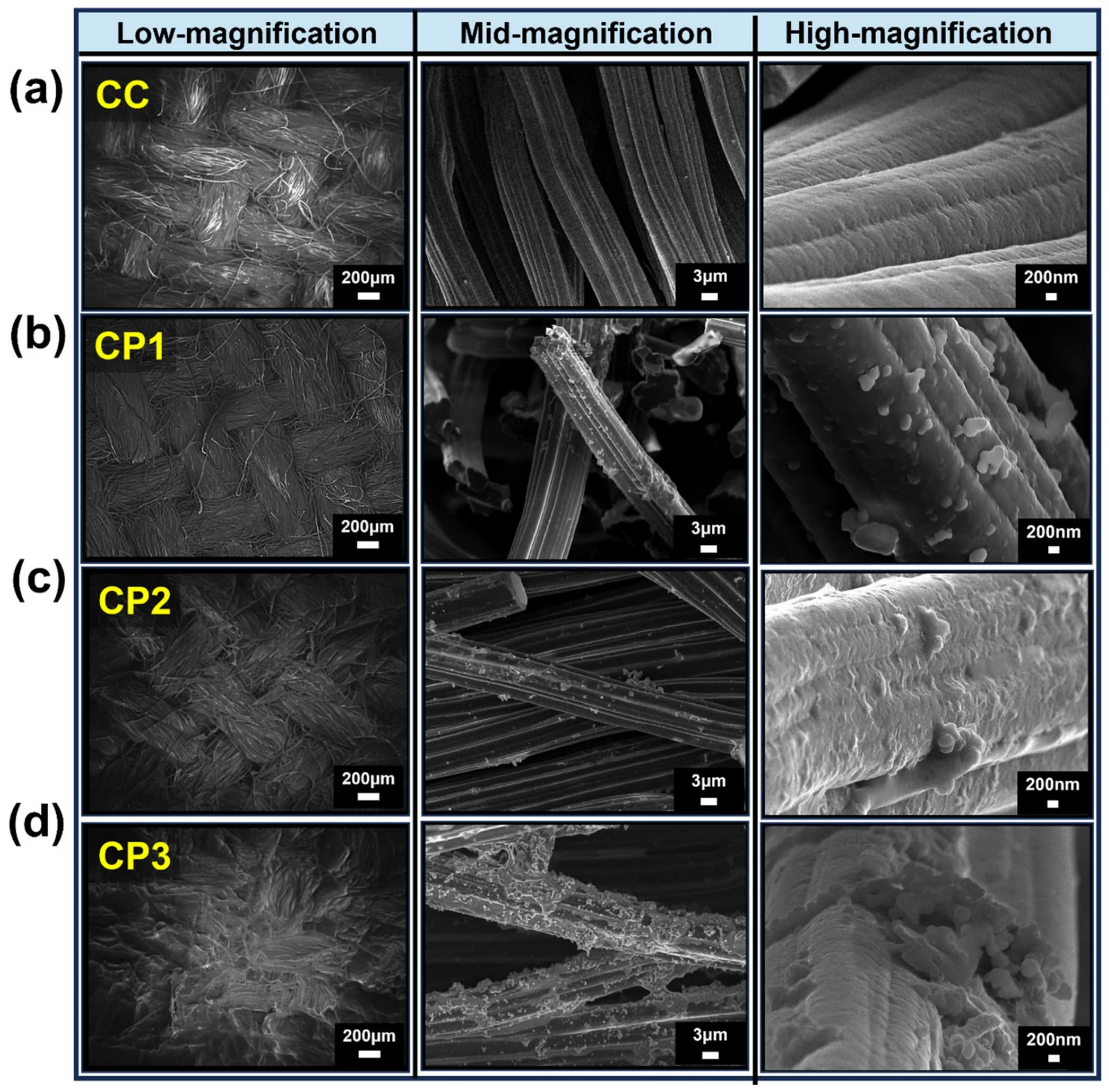
(**a**–**d**) SEM images of CC and CC/PPy composites with different polymerization times at low, intermediate, and high magnifications.

Figure 2a presents SEM images of pristine CC. At low magnification, a woven architecture consisting of interlaced carbon fibers is clearly observed. At intermediate and high magnifications, individual carbon fibers exhibit relatively smooth, well-defined, and uniform surfaces [18]. Figure 2b shows SEM images of CP1. At intermediate magnification, PPy particles are attached to the carbon fiber surface in a particulate manner, indicating discontinuous PPy layer formation during the initial nucleation stage. At high magnification, PPy particles distributed across the fiber surface are clearly observed. Figure 2c presents SEM images of CP2. Compared with CP1, the surface coverage is increased, and PPy forms a more continuous and uniform layer along the fiber surface. Figure 2d shows SEM images of CP3. At intermediate magnification, excessive PPy growth along the fibers is evident, and at high magnification, overgrowth-induced agglomerates are observed, resulting in higher surface roughness and lower coating uniformity. These results demonstrate that increasing the polymerization time generally promotes PPy growth and increases the extent of fiber-surface coverage [19]. However, the coating morphology varies markedly with polymerization time. In CP1, the short polymerization time leads to discontinuous, particulate PPy growth corresponding to the initial nucleation stage, whereas CP3 exhibits localized agglomeration caused by excessive PPy growth during prolonged polymerization. In contrast, CP2 shows the most continuous and relatively uniform PPy coating morphology among the investigated samples.

**Figure 3:**
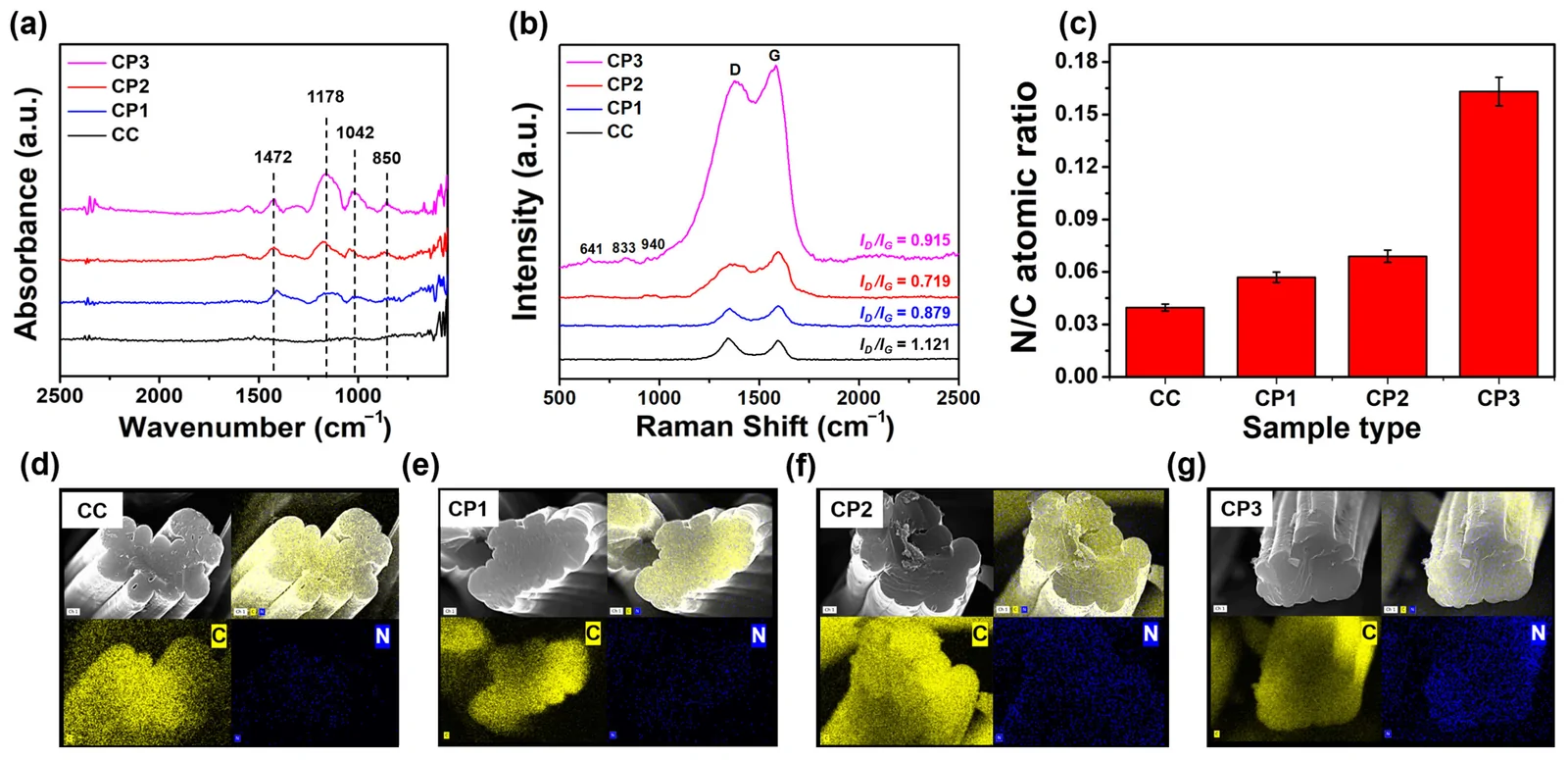
(**a**) FT-IR spectra, (**b**) Raman spectra, and (**c**) N/C atomic ratio quantified from EDS analysis of CC and CC/PPy composites (CP1–CP3) prepared with different polymerization times. (**d**–**g**) Cross-sectional SEM–EDS elemental mapping images of C and N for CC, CP1, CP2, and CP3, respectively, confirming the distribution of nitrogen throughout the fiber cross-section.

PPy formation as a function of polymerization time was examined by FT-IR and Raman spectroscopy. Figure 3a shows the FT-IR spectra of pristine CC and the CC/PPy composites. Pristine CC exhibits no prominent, well-defined peaks, showing only weak and broad absorption bands. In contrast, the CC/PPy composites exhibit characteristic PPy-related peaks. Specifically, the band at ~850 cm^−1^ is assigned to C–H vibration, the band at ~1042 cm^−1^ is associated with N–H vibration, and the band at ~1178 cm^−1^ corresponds to vibrations of the pyrrole ring. In addition, the band at ~1472 cm^−1^ is related to C–N vibration. Overall, the intensities of the PPy-related peaks increase with increasing polymerization time, indicating an increased contribution of PPy within the CC/PPy composites [20].

Figure 3b presents the Raman spectra of pristine CC and the CC/PPy composites. All samples exhibit the D band (~1350 cm^−1^) and G band (~1580 cm^−1^), corresponding to disordered carbon domains and the C=C stretching vibration of sp^2^-bonded carbon, respectively [21,22]. With increasing polymerization time, the spectral features in the D- and G-band region become more pronounced, reflecting an increased contribution from PPy to the composite spectra. In addition to the carbon-related D and G bands, weak PPy-related Raman features are also observed in the CC/PPy composites. In particular, CP3 exhibits weak peaks at approximately 641, 833, and 940 cm^−1^, which can be assigned to pyrrole-ring deformation, C–H deformation, and ring deformation associated with doped PPy, respectively. Meanwhile, the major Raman bands of PPy, including C–N/C=C backbone and ring-stretching vibrations, mainly appear in the 1300–1600 cm^−1^ region [23] and therefore strongly overlap with the D and G bands of the underlying carbon cloth.

The variation in the *I_D_*/*I_G_* ratio provides additional information on the structural evolution of the CC/PPy composites. Pristine CC exhibits the highest *I_D_*/*I_G_* ratio because its fibrous surface is relatively defect-rich and disordered. After PPy polymerization, the CC/PPy composites show decreased *I_D_*/*I_G_* ratios, indicating that PPy deposition reduces the relative contribution of defect-related signals from the underlying carbon cloth. In particular, CP2 shows the lowest *I_D_*/*I_G_* ratio (0.719), suggesting that under this polymerization condition, PPy was formed most continuously and relatively uniformly on the fiber surface. In contrast, the *I_D_*/*I_G_* ratio of CP3 increases again to 0.915. This re-increase is attributed to excessive PPy growth during prolonged polymerization, which may induce localized nucleation and agglomerative growth. Such overgrowth can reduce coating uniformity and disturb PPy chain alignment and π–π stacking, thereby increasing structural disorder [24]. At the same time, the overall Raman intensity of CP3 becomes significantly stronger because prolonged polymerization increases the amount of PPy deposited on the CC surface, thereby increasing the contribution of PPy-related Raman features. This interpretation is also consistent with the FT-IR results, in which the PPy-related characteristic peaks become more pronounced with increasing polymerization time, and with the SEM observations in Figure 2, where CP3 exhibits increased surface roughness and more aggregated PPy domains than CP2. Therefore, the high Raman intensity and re-increased *I_D_*/*I_G_* ratio observed for CP3 indicate that prolonged polymerization promotes further PPy deposition, but simultaneously leads to a thicker, less uniform, and more structurally disordered PPy coating.

Figure 3c summarizes the N/C atomic ratio of CC and the CC/PPy composites as a function of polymerization time, calculated from EDS results. Pristine CC shows the lowest N/C ratio (0.04) due to its carbon-based framework. When normalized to only C and N, this corresponds to an N content of ~3.8 at%, which is close to the detection-limit-level trace N signal in EDS. Such a weak N signal may originate from residual nitrogen species associated with the carbon fiber manufacturing process or trace contamination introduced during sample preparation. In contrast, the N/C ratios of CP1 and CP2 increase to 0.06 and 0.07, respectively. When the elemental composition is normalized considering only carbon and nitrogen, these values correspond to approximately 5.7 at% and 6.5 at% nitrogen. CP3 exhibits the highest N/C ratio of 0.16, corresponding to an increased N content of ~13.8 at%. These results qualitatively suggest that the amount of nitrogen-containing species in the CC/PPy composites increases with polymerization time. This trend is consistent with the increased PPy-related contribution indicated by the FT-IR, and Raman results.

Figure 3d–g show the elemental distributions of CC and the CC/PPy composites obtained from cross-sectional SEM–EDS mapping. For pristine CC, the N signal is barely detectable, and C is confirmed as the dominant element. In contrast, the CC/PPy composites exhibit a clear N signal, confirming the increased nitrogen content after PPy polymerization. Importantly, the N signal is observed across the cross-section rather than being confined to the outer surface, suggesting that nitrogen-containing species are distributed throughout the fibrous structure. Furthermore, the relative intensity of the N signal increases with polymerization time, indicating an increased incorporation of nitrogen-containing species in the composites. These observations suggest that pyrrole vapor can diffuse into the porous fibrous structure during VPP, leading to the presence of nitrogen-containing species throughout the carbon-cloth cross-section [25]. However, because EDS mapping alone cannot unambiguously distinguish PPy from residual nitrogen-containing species, the cross-sectional N distribution is not interpreted as direct proof of PPy penetration. Instead, it is used only as supporting evidence for the distribution of nitrogen-containing species within the porous carbon-cloth structure.

**Figure 4:**
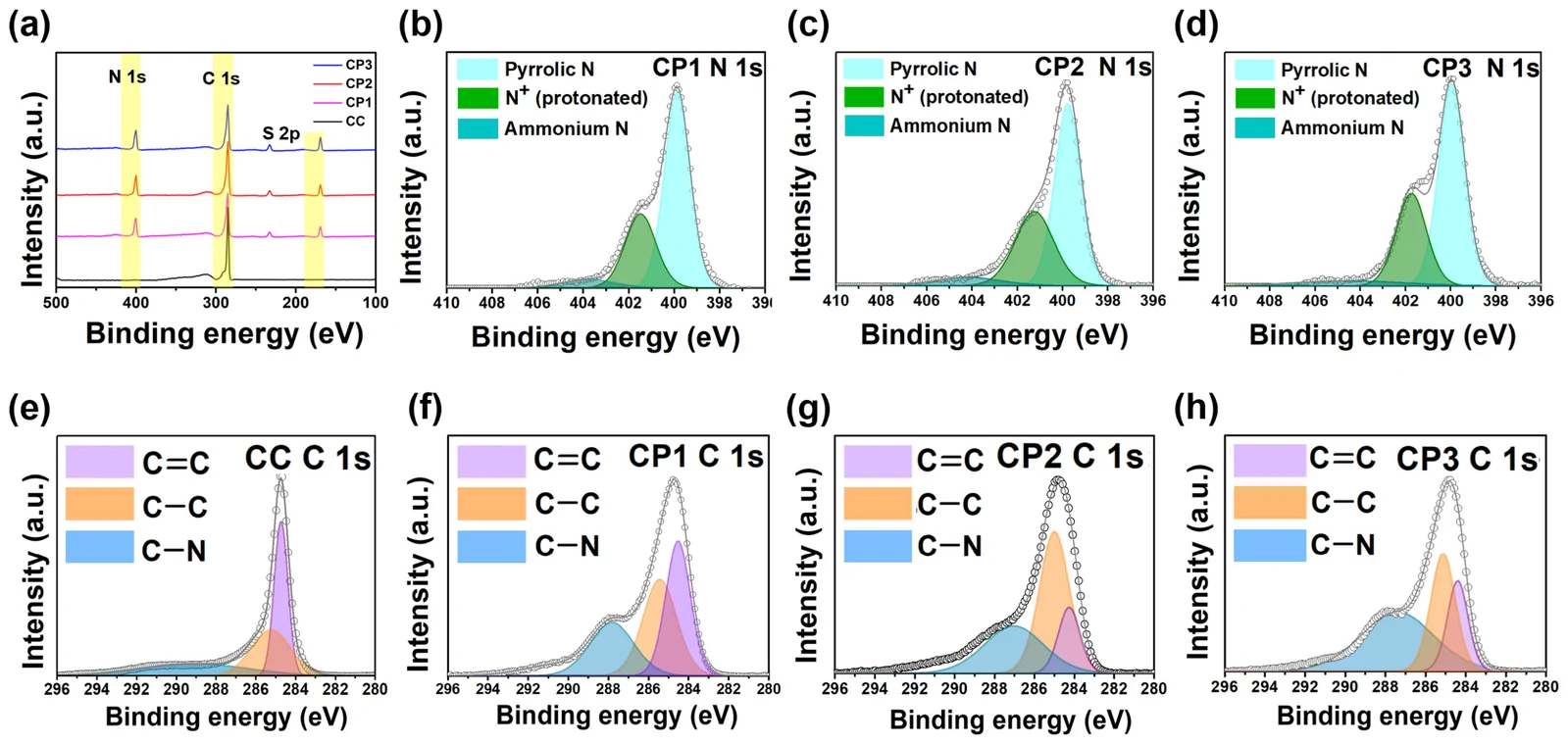
(**a**) XPS survey spectra of CC and CC/PPy composites with different polymerization times, confirming the successful formation of PPy. (**b**–**d**) High-resolution N 1s spectra of CC/PPy composites (CP1–CP3) as a function of polymerization time, showing changes in nitrogen bonding configurations. (**e**–**h**) High-resolution C 1s spectra of CC and CC/PPy composites, revealing the evolution in carbon bonding configurations upon PPy formation.

To gain further insight into the chemical bonding states of the CC/PPy composites, XPS analysis was performed, and the results are presented in Figure 4. Figure 4a presents the XPS survey spectra obtained from the top few nanometers of the surface. For pristine CC, the C signal is dominant (95.21 at%) with a minor O contribution (4.09 at%), while N and S are below the detection limit. In contrast, CP1–CP3, which incorporate PPy, exhibit newly emerged N and S signals that are absent in pristine CC, confirming the formation of PPy as well as the presence of APS-derived sulfur species. The N content remains comparable across CP1–CP3, ranging from 11.82 to 12.30 at%. Meanwhile, the S content gradually increases with polymerization time, from 5.8 at% for CP1 and 5.9 at% for CP2 to 6.79 at% for CP3. This suggests that the surface N content becomes relatively insensitive to further polymerization once the surface is rapidly covered by PPy at the early stage of VPP, whereas longer polymerization promotes greater incorporation of APS-derived sulfur species. Since XPS probes only the top few nanometers, the N 1s signal of pristine CC is near the detection limit, making reliable peak deconvolution and quantitative fitting challenging. Accordingly, high-resolution N 1s analysis was conducted only for the CC/PPy composites.

Figure 4b–d show the high-resolution XPS spectra in the N 1s region. In general, PPy formed via VPP consists of neutral pyrrolic N along with positively charged nitrogen species (N^+^) generated during doping [26]. The peak at ~399.86 eV is assigned to pyrrolic N associated with the –NH moiety in the PPy backbone, whereas the peak at ~401.5 eV corresponds to protonated N^+^ species, which are directly related to the doped state of PPy. In addition, a weak broad component observed near 404 eV was tentatively assigned to ammonium-related nitrogen species, likely associated with residual APS-derived species, since APS was used as the oxidizing agent in this study [27].

Table 1 summarizes the nitrogen bonding compositions of the CC/PPy composites as a function of polymerization time. CP1 exhibits the highest pyrrolic N fraction (64.7%) and the lowest protonated N^+^ fraction (29.6%), indicating that under the shortest polymerization time, PPy growth and oxidation/doping are relatively limited, leaving a larger fraction of the polymer in a neutral state [28]. In contrast, CP2 shows the lowest pyrrolic N fraction (58.3%) and the highest protonated N^+^ fraction (34.9%), suggesting the most favorable doping state among the investigated samples. This result indicates that under this condition, VPP proceeds sufficiently to form a well-developed and effectively doped PPy layer. With a further increase in polymerization time, CP3 shows an increase in pyrrolic N fraction and a decrease in protonated N^+^ fraction. This behavior suggests that prolonged PPy growth does not necessarily lead to a continuous increase in the effective doping level of the PPy backbone. As polymerization proceeds, the PPy layer becomes thicker and more structurally heterogeneous, as evidenced by the agglomerated morphology observed for CP3. Under these conditions, oxidant transport and reaction within the growing polymer layer may become diffusion-limited and spatially non-uniform, which can result in heterogeneous oxidation and doping states across the PPy layer [29]. Because XPS mainly reflects the near-surface composition, such heterogeneity may be manifested as an increased pyrrolic N fraction and a decreased protonated N^+^ fraction in CP3. Importantly, the increased sulfur content in CP3 does not necessarily indicate a higher effective doping level of the PPy backbone, because the XPS sulfur signal can include residual APS-derived sulfur species in addition to sulfur species directly involved in charge compensation. Therefore, the increased sulfur content and decreased protonated N^+^ fraction in CP3 indicate that the total amount of APS-derived sulfur species and the effective doping state of PPy do not necessarily vary proportionally.

The ammonium-related component near 404 eV contributes only a minor fraction (approximately 5–7%) in all samples. Therefore, this component was not considered an intrinsic nitrogen state directly involved in charge transport within the PPy backbone, and the discussion of electrical-property variation was focused primarily on the relative changes in pyrrolic N and protonated N^+^.

Figure 4e–h show the high-resolution XPS spectra in the C 1s region. The peak at ~284.39 eV corresponds to C=C bonds in sp^2^ carbon, originating from the graphitic carbon framework of CC. The component at ~285.15 eV is generally assigned to C–C bonds, reflecting contributions from non-conjugated carbon species in the PPy formed on the CC surface. It also includes contributions from surface defects and amorphous carbon components on the CC surface. The peak at ~287.41 eV falls within the region where C–N and C–O contributions can overlap; in the present composites, this component likely includes C–N bonds from PPy together with possible C–O species formed during oxidation. Consequently, the C 1s results show that, upon PPy formation, the relative contribution of sp^2^ carbon (C=C) from CC decreases, while the contributions from PPy- and oxidation-related components (C–C and C–N/C–O) increase [30].

Table 2 summarizes the carbon bonding states of CC and the CC/PPy composites, highlighting how the carbon bonding composition changes upon PPy formation. Compared with pristine CC, the CC/PPy composites exhibit an overall decrease in the sp^2^ carbon (C=C) fraction, which is attributed to the reduced relative contribution from the underlying CC graphitic signal due to PPy coverage. In contrast, the C–N/C–O fraction increases with increasing polymerization time, suggesting not only an increased contribution of C–N bonds from PPy but also a growing contribution of oxidation-induced C–O species. Notably, the C–N/C–O fraction increases from 33.2% in CP2 to 47.5% in CP3, corresponding to an increase of approximately 14.3%. This increase may reflect both the increased PPy content (higher C–N contribution) and additional formation of oxygen-containing surface functionalities (C–O) due to prolonged exposure to an oxidative environment at longer polymerization times. In CP2, the decreased C=C fraction and the markedly increased C–C fraction suggest that PPy forms with high surface coverage under this condition. As a result, the relative sp^2^ signal from the substrate is reduced, while the C–C contribution from PPy formation is enhanced. The continuous PPy morphology observed in the SEM image in Figure 2c provides supporting evidence for this interpretation.

**Figure 5:**
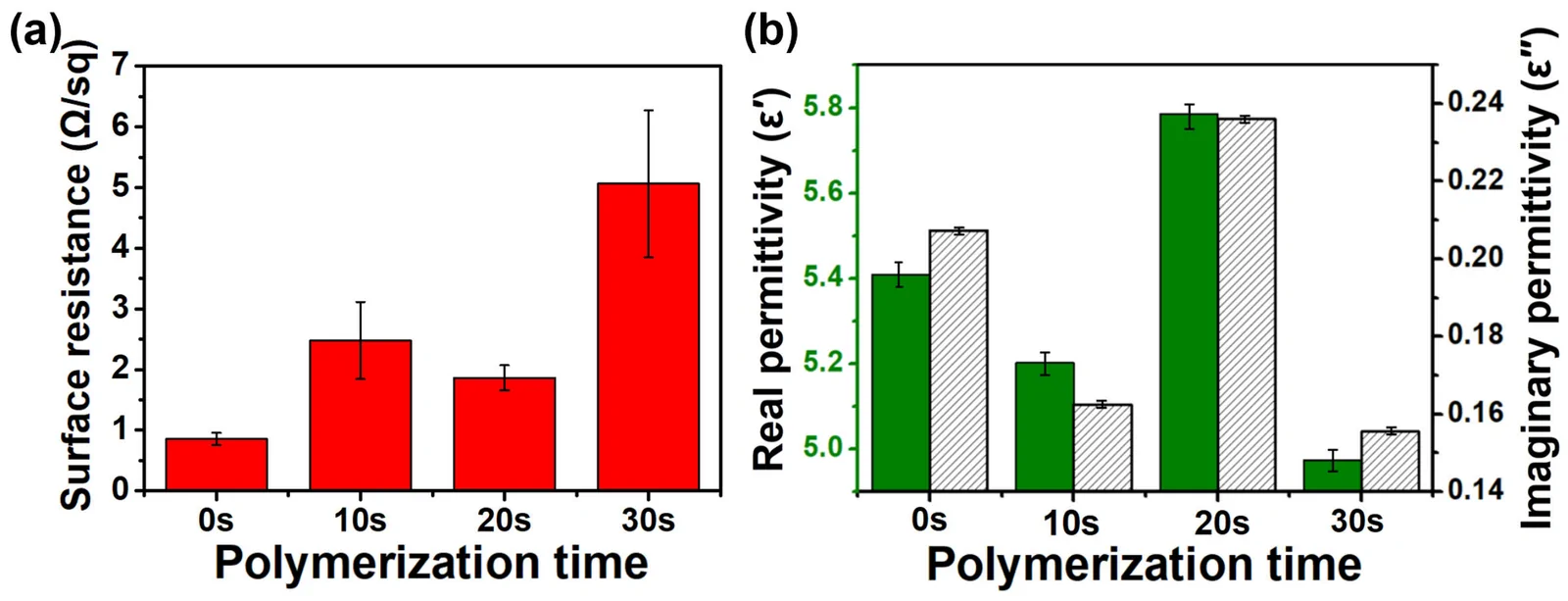
(**a**) Surface resistance and (**b**) real (ε′) and imaginary (ε″) parts of the complex permittivity of CC and CC/PPy composites prepared with different polymerization times.

Figure 5a shows the variation in surface resistance of pristine CC and the CC/PPy composites as a function of polymerization time. Charge transport in the CC/PPy composites is governed not only by continuous conductive pathways within the PPy layer but also by inter-domain charge transfer between discontinuously distributed PPy domains via hopping and tunneling. Accordingly, surface resistance is collectively influenced by the continuity of PPy formation and domain connectivity, contact resistance at domains and interfaces, and the carrier-transport barriers formed by the doping level as well as structural defects and disorder [31]. Pristine CC shows a surface resistance of 0.86 Ω/sq. Among the composites, CP1 exhibits a relatively high surface resistance of 2.48 Ω/sq, which is attributed to insufficient PPy growth at the shortest polymerization time, resulting in a discontinuous coating. In this case, PPy is distributed as localized island-like domains, which increases the inter-domain spacing and reduces the effective contact area. As a result, the probability of inter-domain charge transfer decreases, and a continuous conduction pathway is not sufficiently established. CP3 also shows a markedly increased surface resistance of 5.06 Ω/sq, which is attributed to non-uniform PPy growth and agglomeration induced by excessive polymerization. This increases microstructural inhomogeneity and elevates interfacial contact resistance and charge-transport barriers. In contrast, CP2 shows a relatively low surface resistance of 1.86 Ω/sq among the composites, because PPy forms a more uniform and continuous layer under this condition. This improves domain connectivity and effective contacts, thereby reducing contact resistance and charge-transport barriers and enabling the most efficient conductive network.

Figure 5b compares the complex permittivity of pristine CC and the CC/PPy composites. The dielectric response of a lossy medium can be expressed as the complex permittivity, ε* = ε′ − jε″. Here, ε′ represents the energy-storage term, which can influence the effective wavelength, resonance condition, and input-impedance formation of RF structures. In contrast, ε″ represents the loss term and is affected by both polarization-relaxation loss and conduction loss associated with charge carriers [32]. For carbon-fiber-based materials such as CC, the complex permittivity has been reported to exhibit strong frequency dispersion, decreasing with increasing frequency, indicating a highly frequency-sensitive dielectric response [33]. Therefore, the complex permittivity measured using an LCR meter in this study was not interpreted as the absolute dielectric property at the antenna operating frequency. Instead, it was used only for the relative comparison of the dielectric responses of the samples under identical measurement conditions. Within this limited comparison, CP2 exhibited the highest values of both ε′ (5.78) and ε″ (0.23) among the investigated samples.

The increased ε′ of CP2 can be explained by an enhancement of Maxwell–Wagner–Sillars (MWS) interfacial polarization. Pristine CC has a porous architecture composed of carbon fibers and air gaps, providing numerous CC/air interfaces where space-charge accumulation can occur under an applied electric field, leading to interfacial polarization. In CC/PPy composites, a three-phase heterogeneous structure (CC/PPy/air) is formed. When charge accumulation is promoted at interfaces between phases with dissimilar electrical properties, MWS polarization can be strengthened, resulting in an increased ε′. For CP1 with a short polymerization time, PPy is discontinuously distributed on the fiber surface, leading to insufficient connectivity between conductive domains; consequently, the formation of effective polarization sites for interfacial charge accumulation is limited. In contrast, CP3 tends to exhibit excessive PPy growth accompanied by agglomerate formation and partial pore filling, which can reduce the effective interfacial area and hinder interfacial charge accumulation [34]. Accordingly, in CP1 and CP3, the contributions from interfacial polarization and conduction loss may not be effectively developed compared to CP2, resulting in lower ε′ and ε″ than those of CC. These interpretations are supported by SEM observations showing discontinuous formation of PPy domains in CP1 and agglomeration with reduced porosity in CP3. By comparison, CP2 exhibits a relatively uniform distribution of PPy domains along the fiber surface while preserving the textile pore structure, thereby providing extended interfaces where conductive domains and insulating air gaps coexist. Together with the low surface resistance, these features facilitate charge transport toward heterogeneous interfaces and promote charge accumulation, thereby maximizing interfacial polarization and yielding the highest ε′. The increase in ε″ can also reflect conduction loss in addition to dielectric loss associated with polarization relaxation. For conductive media, the conduction-loss term is known to contribute to ε″ and can be approximated as ε″ ≈ σ/(ωε_0_) [35], where σ is the electrical conductivity and ω is the angular frequency. Considering the low surface resistance observed in Figure 5a, the highest ε″ in CP2 is more likely attributed to an increased contribution of conduction loss arising from the development of a conductive network, rather than to an increase in polarization-relaxation loss.

**Figure 6:**
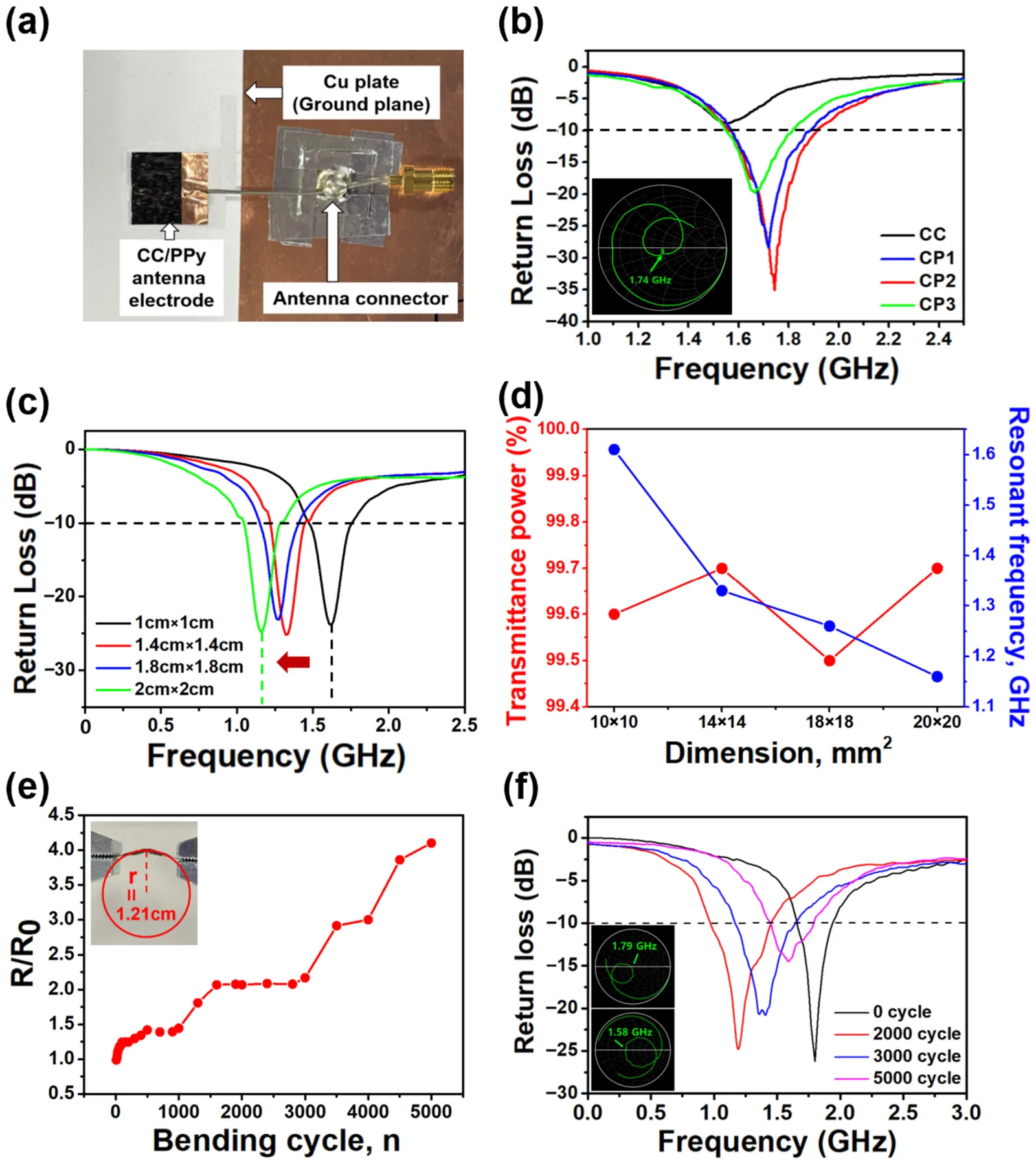
(**a**) Photograph of a CC/PPy composite-based monopole patch RF antenna with a rectangular Cu ground plane. (**b**) RL curves of CC/PPy composite-based antennas with different polymerization times. The inset shows the Smith chart of the input impedance, indicating the impedance matching behavior. (**c**) RL curves of CC/PPy composite-based antennas with different electrode dimensions. (**d**) Transmittance power and resonance frequency as a function of electrode dimension. (**e**) Normalized resistance change (*R*/*R*_0_) of the CC/PPy composite-based antenna as a function of bending cycles, measured at a bending radius of 1.21 cm, where *R*_0_ is the surface resistance before bending. (**f**) RL curves of the CC/PPy composite-based antenna as a function of bending cycles. The inset shows the Smith chart impedance diagrams of the antenna before bending (0 cycles) and after 5000 bending cycles.

Figure 6 summarizes the overall characteristics of the monopole patch antennas based on CC and the CC/PPy composites. In general, antenna performance is primarily evaluated in terms of the center frequency, bandwidth, and RL. In addition, the electrical stability under repeated bending was analyzed. Figure 6a illustrates the measurement configuration. A rectangular Cu plate was used as the ground plane, and the antenna electrode of the CC/PPy composite was connected to the measurement system via an SMA connector to ensure stable signal transmission [36].

Figure 6b presents the measured RL of the antennas based on CC and the CC/PPy composites. RL is a metric that quantifies the power loss arising from impedance mismatch; minimizing RL is essential for optimizing power transfer. The return loss is generally expressed as RL (dB) = 10 log_10_ (*P_r_*/*P_i_*), where *P_i_* and *P_r_* are the incident and reflected powers, respectively [37]. Compared with pristine CC, the CC/PPy composite-based antennas exhibit an overall reduction in RL, indicating improved impedance matching. The input impedance can be expressed as Z_in_ = R + jX, where R is the real component including radiation and loss resistances, and X is the reactive component governed by stored energy. Because standard RF measurement systems are referenced to 50 Ω, reflection is reduced as Z_in_ ≈ 50 Ω. The minimum RL values of CP1, CP2, and CP3 were −28.26, −34.81, and −19.6 dB, respectively, and CP2 showed the lowest RL, suggesting that the most favorable matching condition was formed for this sample.

This RL behavior can be interpreted together with the surface resistance results in Figure 5a. The real part of the input impedance can be written as R_rad_ + R_loss_, where R_rad_ is radiation resistance and R_loss_ represents losses originating from the conductor and dielectric [38]. A lower surface resistance enhances conductivity and can reduce conductor-related dissipation during current flow, thereby decreasing R_loss_, whereas a higher surface resistance can increase R_loss_. In agreement with this trend, CP2 exhibited the lowest surface resistance among the composites and the minimum RL. By contrast, CP1 showed less improvement due to insufficient conductive pathway development at short polymerization time, and CP3 showed degraded matching resulting from increased surface resistance under excessive polymerization. These results indicate that conductivity-driven changes in loss components and current distribution contribute to the observed input-impedance matching.

The permittivity characteristics in Figure 5b are also relevant to impedance formation and resonance. The real permittivity ε′ is associated with electric-field energy storage and influences the effective wavelength, which alters the resonance condition and the reactive component X. The relatively high ε′ of CP2 may facilitate adjustment of the resonance condition and the reactive term, shifting the impedance trajectory closer to the 50 Ω reference point. The imaginary permittivity ε″ reflects dielectric loss related to polarization relaxation and may also include contributions associated with charge transport. The relatively high ε″ observed for CP2 is qualitatively consistent with the enhanced conduction pathways inferred from the reduced surface resistance. The Smith chart inset in Figure 6b further supports the superior matching of CP2, showing impedance points distributed closer to the chart center. The matching is optimized near the frequency where RL reaches its minimum of −34.81 dB, implying a very small reflected-power fraction and efficient power delivery. The RL minimum is observed at 1.74 GHz, suggesting potential applicability to mobile communications and IoT applications.

Figure 6c shows the resonance frequency and RL of the CC/PPy composite-based monopole patch antenna as a function of electrode dimension. As the electrode size increases, the resonance frequency shifts to lower values, which is consistent with the general antenna behavior that a larger radiating element increases the effective electrical length and thus lowers the resonance frequency [39]. Notably, RL remains below −10 dB despite the frequency shift, demonstrating that the resonance frequency and matching condition can be effectively tuned by adjusting the electrode dimension without an additional impedance-matching circuit. Figure 6d summarizes the resonance frequency together with the transmittance power under the same conditions. The transmittance power represents the fraction of input power delivered to the antenna after excluding the reflected component. The transmittance power remains above 99% for all electrode sizes, indicating that the electrode-dimension-induced frequency tuning is achieved while maintaining favorable impedance matching. Meanwhile, the resonance condition is governed by the effective wavelength, which decreases as the effective permittivity increases. Therefore, under conditions with higher effective permittivity, the required electrical length at a given operating frequency can be reduced, which is advantageous for antenna miniaturization [40]. In this context, considering that CP2 exhibits a relatively high ε′ in Figure 5b, the dielectric characteristics of the composite may contribute to tuning the electrical length and enabling potential miniaturization.

Figure 6e presents the normalized surface resistance (*R*/*R*_0_) of the CC/PPy electrode as a function of bending cycles, where R_0_ denotes the surface resistance before bending. The *R*/*R*_0_ value increased continuously with increasing cycle number and reached approximately 4.5 after 5000 cycles, indicating degradation of the electrical characteristics during repeated mechanical deformation. The absolute surface resistance increased from 1.86 to 8.37 Ω/sq after 5000 bending cycles. SEM observations before and after the bending test showed that the overall woven fiber-bundle architecture was largely maintained after repeated bending, with no obvious large-scale fracture, fiber-bundle breakage, severe surface cracking, or apparent large-area PPy delamination (Figure S2). These observations suggest that the increase in surface resistance was not primarily caused by catastrophic structural failure at the electrode level. Instead, repeated bending may have induced subtle rearrangement or slippage of the interwoven fiber bundles, together with changes in fiber-to-fiber and yarn-to-yarn contact resistance, which could contribute to the observed resistance increase [41,42]. Additionally, the environmental stability of the CC/PPy electrode was evaluated under accelerated high-humidity conditions (40 °C and 80 ± 5% RH). The surface resistance increased gradually with exposure time, reaching an R/R0 value of 1.82 after 48 h, but the electrode remained conductive throughout the test period, indicating that prolonged exposure to high humidity affects the electrical characteristics of the electrode without causing complete loss of conductivity (Figure S3).

Figure 6f shows the evolution of RL with bending cycles. The RL changed from −26.3 dB at 0 cycle to −24.8, −20.6, and −14.4 dB after 2000, 3000, and 5000 cycles, respectively, indicating gradual degradation of the impedance-matching condition with repeated bending. Nevertheless, the RL remained below −10 dB even after 5000 cycles, confirming that the antenna remained operational after repeated mechanical deformation. This degradation in RL can be attributed to bending-induced changes in the electrical characteristics of the radiating electrode and the resulting shift in the impedance-matching condition. The inset Smith charts support this interpretation: the 0-cycle condition shows an impedance locus located close to the 50 Ω center near resonance, whereas the locus after 5000 cycles deviates farther from the 50 Ω reference point, confirming the change in matching condition and the accompanying increase in RL.

**Figure 7:**
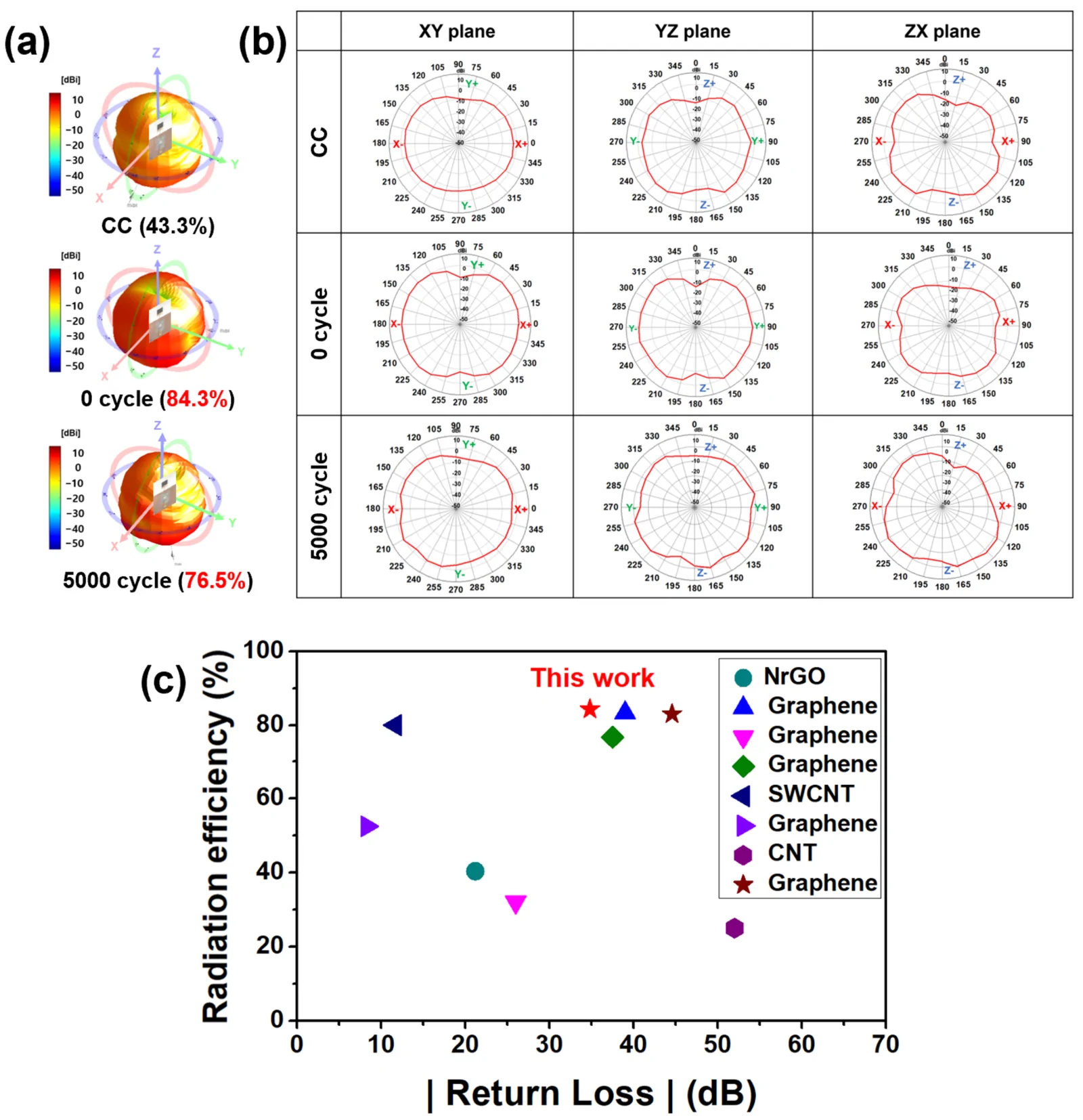
(**a**) Measured 3D radiation patterns and (**b**) 2D radiation patterns in the XY, YZ, and ZX planes of CC and the CC/PPy composite-based antenna (CP2). (**c**) Comparison of return loss (|S_11_|) and radiation efficiency for the proposed CC/PPy antenna and representative carbon-based flexible RF antennas reported in the literature.

The radiation efficiency, peak gain, and peak directivity were further analyzed to evaluate the radiation characteristics. Figure 7a shows the 3D radiation patterns of the monopole patch RF antennas based on CC and the CC/PPy composites. Radiation efficiency is defined as the ratio of the total radiated power to the input power and is a key metric representing the RF transmit/receive efficiency of an antenna. Peak gain represents the radiation intensity in a specific direction relative to an isotropic radiator and thus reflects directional behavior. The antenna characteristics are summarized in Table 3. Compared with CC, the CC/PPy composite-based antennas (particularly under the CP2 condition) exhibit excellent radiation performance, achieving a peak gain of 3.30 dBi, a peak directivity of 3.91 dBi, and a radiation efficiency of 84.32%. Although RL reflects the impedance-matching condition and the fraction of reflected power, radiation efficiency additionally depends on how effectively the accepted power is converted into radiated power rather than being dissipated within the electrode structure. Therefore, the superior radiation efficiency of CP2 is considered to result not only from its improved impedance matching, but also from the more favorable RF current pathways and more efficient utilization of the accepted power enabled by the relatively uniform PPy coating. Furthermore, after the 5000-cycle bending test, the radiation efficiency remained at 75.61%, indicating that the antenna preserved acceptable radiation performance despite the increase in surface resistance during cyclic bending. These results demonstrate that the CC/PPy composite is a promising flexible electrode material for high-performance monopole patch RF antennas.

Figure 7b presents the corresponding 2D radiation patterns obtained by slicing the 3D patterns in the vertical and horizontal planes. The CP2-based CC/PPy antenna exhibits quasi-omnidirectional radiation with relatively uniform emission in all directions, which is desirable for various RF applications. The concave region observed in the XY plane was affected by the connection condition between the antenna and the adapter during radiation-efficiency measurement. The concave regions observed in the YZ and ZX planes arise from interference at specific angles due to reflected components between the antenna and the ground plane [43]. Compared with CC, CP2 shows overall more uniform radiation characteristics. In the XY plane, CP2 exhibits reduced asymmetry relative to CC, approaching a more omnidirectional pattern, and the pattern distortion in the YZ and ZX planes is also mitigated. This behavior is consistent with the improved PPy layer uniformity achieved under the CP2 condition, which stabilizes the current distribution and thereby enhances radiation efficiency. Furthermore, after the 5000-cycle bending test, the 2D radiation patterns did not exhibit significant deformation, indicating that the quasi-omnidirectional radiation characteristic is well maintained.

Figure 7c compares the radiation efficiency and absolute |S_11_| values of representative carbon-based antennas (e.g., CC, CNT, graphene, graphene paper, and SWCNT) reported in recent literature [39,44,45,46,47,48,49,50]. The reported performances are broadly distributed, with absolute |S_11_| values ranging from 8.3 to 52 dB and radiation efficiencies ranging from 25 to 83.3%. In comparison, the CC/PPy antenna developed in this study shows competitive performance, combining high radiation efficiency with favorable impedance matching. This behavior is attributed to reduced conductor loss associated with the low surface resistance of the radiating electrode, together with improved impedance matching facilitated by the dielectric characteristics of the composite. Overall, these results indicate that the simultaneous optimization of electrical and dielectric properties is an effective strategy for developing high-performance flexible RF antenna electrodes based on carbon cloth.

## 4. Conclusions

In conclusion, this study demonstrated a strategy for fabricating high-performance flexible RF antenna electrodes by simultaneously optimizing the conductivity and complex permittivity of CC/PPy composites through controlled VPP. By systematically varying the polymerization time, the surface resistance and dielectric properties of the CC/PPy composites were tuned, and the PPy formation mechanism and doping states were elucidated through comprehensive structural and chemical characterization. Under the optimized condition, the antenna achieved a return loss of −34.81 dB and a radiation efficiency of 84.32% at 1.74 GHz, representing one of the highest performances reported for carbon-based flexible antennas. Quasi-omnidirectional radiation behavior was also confirmed, and the antenna maintained a return loss of −14.4 dB and a radiation efficiency of 76.5% even after 5000 bending cycles, demonstrating excellent mechanical reliability. These results validate that simultaneous optimization of conductivity and dielectric properties, rather than conductivity alone, is an effective approach for realizing CC-based flexible RF antenna electrodes and for expanding the functional utility of carbon-cloth-based materials in wearable electronics, IoT devices, and vehicular communication systems.

## Figures and Tables

**Table 1:** Summary of nitrogen bonding compositions of CC/PPy composites determined from XPS analysis. Nitrogen bonding states were derived from the N 1s spectra.

Sample	Nitrogen Bonding Composition (%)		
	Pyrrolic N	N^+^ (Protonated)	Ammonium N
CP1	64.7	29.6	5.7
CP2	58.3	34.9	6.8
CP3	61.6	32.5	5.9

**Table 2:** Summary of carbon bonding compositions of CC/PPy composites determined from XPS analysis. Carbon bonding states were derived from the C 1s spectra.

Sample	Carbon Bonding Composition (%)		
	C=C	C–C	C–N/C–O
CC	41	34.3	24.7
CP1	37.3	36.5	26.3
CP2	16.8	50	33.2
CP3	20.5	32	47.5

**Table 3:** Antenna characteristics of CC and CP2-based monopole patch antennas with dimensions 10 mm × 10 mm.

Sample	Resonant Frequency (GHz)	Bandwidth (MHz)	VSWR	Absolute RL Value (dB)	Transmitted Power (%)	Peak Gain (dBi)	Peak Directivity (dBi)	Radiation Efficiency (%)
CC	1.55	N/A	2.00	8.91	88.9	0.88	2.03	43.25
0 cycles	1.74	350	1.04	34.81	100	3.30	3.91	84.32
5000 cycles	1.59	350	1.48	14.4	96.3	2.75	3.61	76.51

Abbreviations: VSWR, voltage standing wave ratio.

## Data Availability

The original contributions presented in this study are included in the article/Supplementary Material. Further inquiries can be directed to the corresponding author.

## Supplementary Materials

The following supporting information can be downloaded at: https://www.mdpi.com/article/10.3390/polym18172124/s1, Figure S1: Effects of (a) APS immersion time, (b) pyrrole dose, (c) APS concentration, and (d) polymerization temperature on the surface resistance of the CC/PPy composites, with all other processing conditions fixed to those used for CP2; Figure S2: SEM images of the CC/PPy composite electrode (a) before bending and (b) after 5000 bending cycles, showing the woven fiber-bundle morphology before and after repeated mechanical deformation; Figure S3: Surface resistance of the CC/PPy electrode as a function of exposure time under accelerated high-humidity conditions (40 °C, 80 ± 5% RH).
