# Supplementary material for: Vapor-Phase Polymerization of Polypyrrole on Carbon Cloth: Simultaneous Tuning of Surface Resistance and Dielectric Permittivity for High-Performance Flexible RF Antenna Electrodes
Source: Polymers (Basel). 2026 Aug 31;18(17):2124. doi: 10.3390/polym18172124 (PMC13568398; doi:10.3390/polym18172124)
Supplement: Supplementary file 1 [file polymers-18-02124-s001.zip › polymers-4501623-supplementary.pdf]

Supplementary Materials

Vapor-Phase Polymerization of Polypyrrole on Carbon Cloth: Simultaneous Tuning of Surface Resistance and Dielectric Permittivity for High-Performance Flexible RF Antenna Electrodes

Seung Ji Kim [1,†], Se Eun Lee [1,2,†], Kyein Kim [1,2] and Keun-Young Shin [1,2,*]

[1] Department of Materials Science and Engineering, Soongsil University, 369 Sangdo-ro, Dongjak-gu, Seoul 06978, Republic of Korea; tmdwl0130@soongsil.ac.kr (S.J.K.); dltpdms8197@soongsil.ac.kr (S.E.L.); kyeinee@soongsil.ac.kr (K.K.)
[2] Department of Convergence of Energy Policy and Technology, Soongsil University, 369 Sangdo-ro, Dongjak-gu, Seoul 06978, Republic of Korea
* Correspondence: skykek@ssu.ac.kr
† These authors contributed equally to this work.

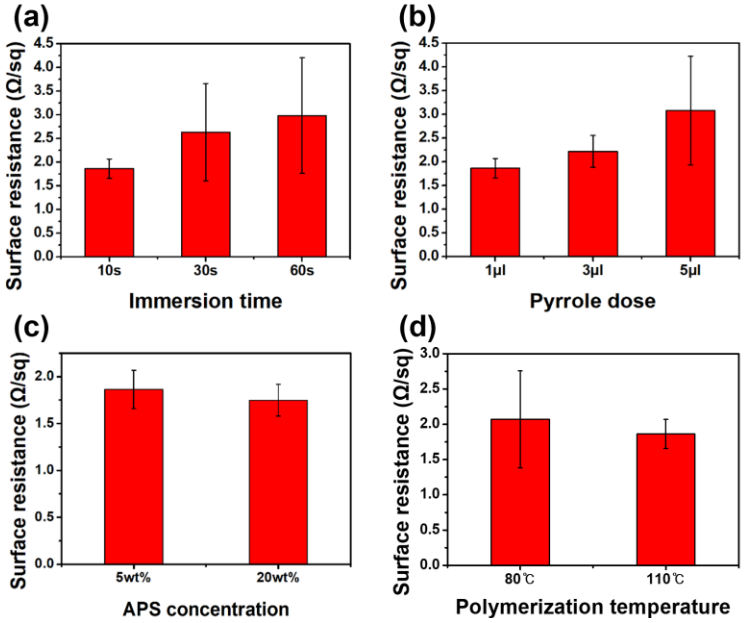

Figure S1. Effects of (a) APS immersion time, (b) pyrrole dose, (c) APS concentration, and (d) polymerization temperature on the surface resistance of the CC/PPy composites, with all other processing conditions fixed to those used for CP2.

Figure S1 summarizes the effects of APS immersion time, pyrrole dose, APS concentration, and polymerization temperature on the surface resistance of the CC/PPy composites. As shown in Figures S1a and S1b, the surface resistance increased with increasing APS immersion time and pyrrole dose. Among the tested conditions, the lowest surface resistance was obtained at an APS immersion time of 10 s and a pyrrole dose of 1 μL. In Figure S1c, the sample prepared with 20 wt% APS exhibited a surface resistance of approximately 1.75 Ω/sq, which was slightly lower than the value of approximately 1.86 Ω/sq obtained with 5 wt% APS. However, the difference between the two conditions was modest, indicating that comparable electrical performance was maintained at 5 wt% APS. Therefore, 5 wt% APS was selected for the subsequent experiments in order to reduce APS consumption while maintaining low surface resistance. In addition, as shown in Figure S1d, the sample polymerized at 110 °C exhibited a slightly lower surface resistance than that polymerized at 80 °C. Therefore, 110 °C was selected as the polymerization temperature for the subsequent experiments because it provided slightly better electrical performance under the present processing conditions.

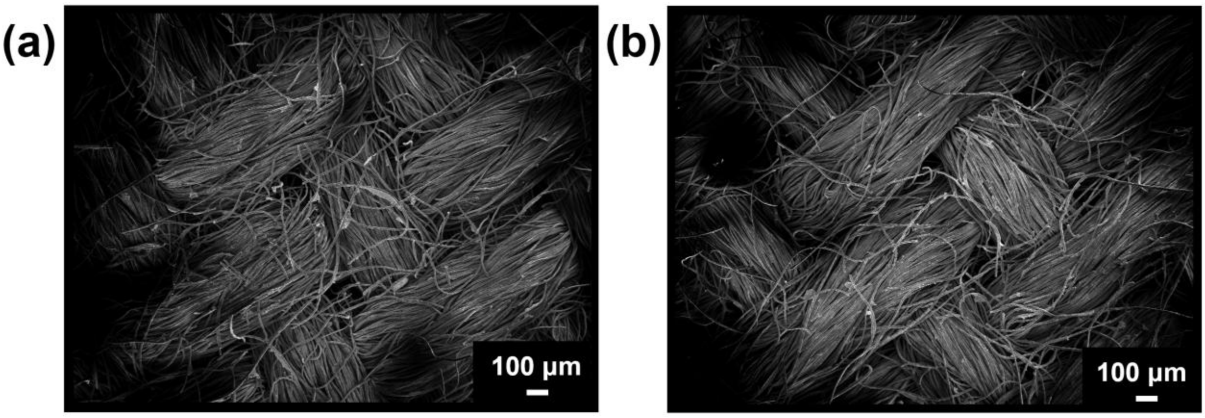

Figure S2. SEM images of the CC/PPy composite electrode (a) before bending and (b) after 5000 bending cycles, showing the woven fiber-bundle morphology before and after repeated mechanical deformation.

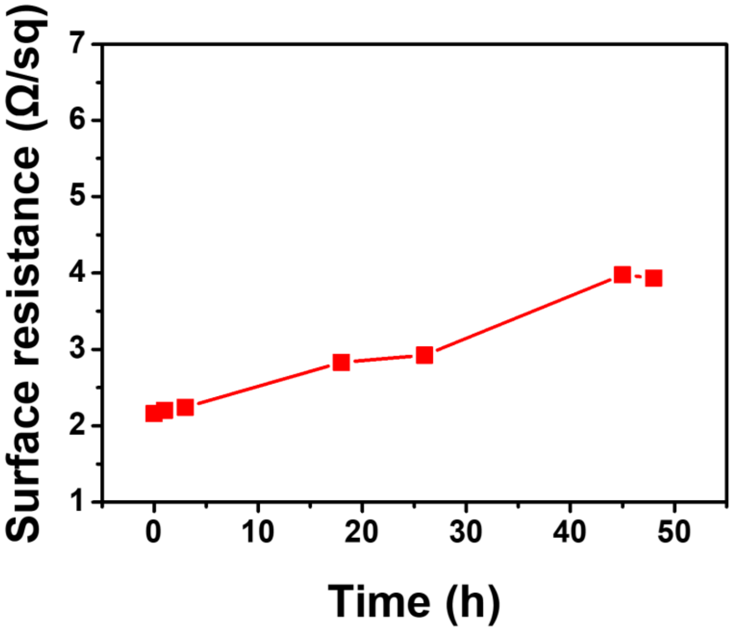

Figure S3. Surface resistance of the CC/PPy electrode as a function of exposure time under accelerated high-humidity conditions (40 °C, 80 ± 5% RH).
